# Resection of high frequency oscillations predicts seizure outcome in the individual patient

**DOI:** 10.1038/s41598-017-13064-1

**Published:** 2017-10-23

**Authors:** Tommaso Fedele, Sergey Burnos, Ece Boran, Niklaus Krayenbühl, Peter Hilfiker, Thomas Grunwald, Johannes Sarnthein

**Affiliations:** 1 University Hospital Zurich, Neurosurgery Department, Zurich, Switzerland; 2 ETH Zurich, Institute of Neuroinformatics, Zurich, Switzerland; 3 Swiss Epilepsy Centre, Zurich, Switzerland; 4 University of Zurich, Zurich Neuroscience Centre, Zurich, Switzerland

## Abstract

High frequency oscillations (HFOs) are recognized as biomarkers for epileptogenic brain tissue. A remaining challenge for epilepsy surgery is the prospective classification of tissue sampled by individual electrode contacts. We analysed long-term invasive recordings of 20 consecutive patients who subsequently underwent epilepsy surgery. HFOs were defined prospectively by a previously validated, automated algorithm in the ripple (80–250 Hz) and the fast ripple (FR, 250–500 Hz) frequency band. Contacts with the highest rate of ripples co-occurring with FR over several five-minute time intervals designated the HFO area. The HFO area was fully included in the resected area in all 13 patients who achieved seizure freedom (specificity 100%) and in 3 patients where seizures reoccurred (negative predictive value 81%). The HFO area was only partially resected in 4 patients suffering from recurrent seizures (positive predictive value 100%, sensitivity 57%). Thus, the resection of the prospectively defined HFO area proved to be highly specific and reproducible in 13/13 patients with seizure freedom, while it may have improved the outcome in 4/7 patients with recurrent seizures. We thus validated the clinical relevance of the HFO area in the individual patient with an automated procedure. This is a prerequisite before HFOs can guide surgical treatment in multicentre studies.

## Introduction

The treatment of choice in patients with drug-resistant focal epilepsy is the surgical resection or disconnection of the epileptogenic zone (EZ)^1^. The EZ is defined as the region indispensable for generating seizures^2^. Recent studies have pointed to the high frequency oscillation (HFO) recorded in intracranial EEG (iEEG) as a new indicator for the EZ^3–5^. HFOs are generally viewed as spontaneous EEG patterns in the frequency range between 80–500 Hz that consist of at least four oscillations that clearly stand out of the background activity^3,5,6^. HFOs are differentiated into “ripples” (80–250 Hz) and “fast ripples” (FRs, 250–500 Hz)^7^. Interictal HFOs have proven more specific in localizing the seizure onset zone (SOZ) than spikes^8^ and have presented a good association with the post-surgery outcome in epilepsy patients^9–12^. While most studies to date only report group results with mean HFO rates in SOZ electrodes exceeding mean rates in non-SOZ electrodes^13^, surgical decisions require the precise classification of cortex sampled by individual electrodes.

To facilitate HFO analysis, several algorithms for their automated or semi-automated detection have been proposed^14–22^. As an advance, we apply here a fully automated definition of HFOs, which we have previously optimized on visual markings in a separate large dataset^22^. While we do not aim to outperform visual marking, we provide a prospective definition of a clinically relevant HFO. We define as HFO area the electrode contacts with the HFO rate exceeding the 95% percentile of the HFO distribution and we focus on ripples co-occurring with FR (FRandR) as a new entity. We quantify the reproducibity of the HFO area over multiple time intervals from different nights of pre-operative monitoring. We evaluate the clinical relevance of HFO in individual electrodes by comparing the HFO area with the resected area (RA) and then by predicting seizure outcome in the individual patient.

## Results

We included 20 patients (Table 1) of whom 9 with mesial temporal lobe epilepsy (TLE) and 11 with extratemporal epilepsy (ETE). Complete seizure freedom (ILAE 1 - International League Against Epilepsy, class 1) was reached in 13 patients, while 7 patients suffered from recurrent seizures (seizure-free rate = 65%).

**Table 1:** Patient characteristics.

Patient	Age, Gender	Histology/Pathology	Epilepsy	Electrode placement	Type of electrodes	Surgery	Nights	Intervals	Test - retest intervals	Test - retest nights	Ripple area resected?	FR area resected?	FRandR area resected?	Outcome (ILAE)	Postoperative follow-up (months)
1	25, M	HS and gliosis	TLE	MTL L, R	5 depth, 1 strip 4 × 1, 1 strip 6 × 1	sAHE; Les	4	28	0.99	1.00	N	N	Y	1	12
2	33, M	Glioma	TLE	MTL L, R	8 depth	sAHE; Les	2	13	1.00	1.00	Y	Y	Y	1	29
3	20, F	HS	TLE	MTL L, R	5 depth	sAHE	5	39	0.78	1.00	N	Y	Y	1	13
4	20, F	HS	TLE	MTL L, R	8 depth	sAHE	6	34	1.00	1.00	N	Y	Y	1	41
5	40, M	HS	TLE	MTL L, R	8 depth	sAHE	5	35	0.94	1.00	Y	Y	Y	1	14
6	48, M	HS	TLE	MTL L, R	8 depth	sAHE	5	35	0.98	1.00	Y	Y	Y	1	11
7	25, M	HS	TLE	MTL L, R	8 depth	sAHE	1	1	—	—	Y	Y	Y	3	42
8	21, F	HS	TLE	MTL L, R	8 depth	sAHE	2	16	0.67	1.00	Y	Y	Y	3	15
9	52, M	HS	TLE	MTL L, R	8 depth	sAHE	2	12	1.00	1.00	Y	Y	Y	5	46
10	37, M	FCD 2b	ETE	Pr R	1 grid 8 × 4; 2 strips 4 × 1	Les	1	6	1.00	—	N	Y	Y	1	36
11	36, M	FCD 2b	ETE	F R	1 grid 8 × 8; 1 depth	Les	3	19	1.00	1.00	Y	Y	Y	1	37
12	49, M	Ganglioglioma	ETE	T-Lat L	1 grid 8 × 4; 1 depth	Les	4	25	0.53	0.67	Y	N	Y	1	25
13	17, M	FCD 1a	ETE	P R	1 grid 8 × 8; 1 depth	Les	2	16	0.75	1.00	N	N	Y	1	25
14	46, F	FCD 1b	ETE	P R	2 grids 8 × 2; 1 strip 6 × 1; 1 strip 4 × 1; 1 depth	Les	2	13	0.97	1.00	Y	Y	Y	1	10
15	31, F	Gliosis	ETE	T-Lat L	1 grid 8 × 4; 2 strips 4 × 1	Les	4	28	0.32	0.33	Y	N	Y	1	25
16	17, F	FCD 2a	ETE	F-Pr-C L	1 grid 8 × 4; 1 depth	Les	2	17	1.00	1.00	N	Y	Y	1	19
17	30, M	FCD 2a	ETE	F R	2 grids 8 × 2; 1 depth	Les	1	1	—	—	Y	Y	N	5	45
18	40, M	FCD 2a	ETE	P R	2 strips 6 × 1; 1 depth	Les	1	5	1.00	—	N	Y	N	5	30
19	38, M	Cavernoma	ETE	T P	1 grids 8 × 4; 1 grids 8 × 2	Les	2	13	0.36	0.00	N	N	N	6	11
20	17, M	FCD 3	ETE	O T	1 grids 8 × 2	Les	4	29	0.23	1.00	N	N	N	5	16

Abbreviations: C = central; depth = depth electrode; ETE = extratemporal lobe epilepsy; F = frontal; FCD = focal cortical dysplasia; FR = fast ripple; FRandR = FR occurring together with ripple; grid = grid electrode; HFO = high frequency oscillation; HS = hippocampus sclerosis; ILAE = International League Against Epilepsy; MTL = mesial temporal lobe; L = left; Lat = lateral; Les = lesionectomy; O = occipital; P = parietal; Pr = precentral; R = right; sAHE = selective amygdala-hippocampectomy; strip = strip electrode; T = temporal; TLE = mesial temporal lobe epilepsy. Test-retest intervals/nights: percentage of scalar products between pairs of intervals/nights exceeding the significance threshold (see Methods: Test-retest reliability of the spatial distribution of HFO). Over the intervals of TLE patients, the median was >98% irrespective of outcome. Over the intervals of ETE patients, the median was 97% for patients with good outcome and only 36% for patients with poor outcome.

### The HFO area predicted seizure outcome in the individual patient

**Figure 1:**
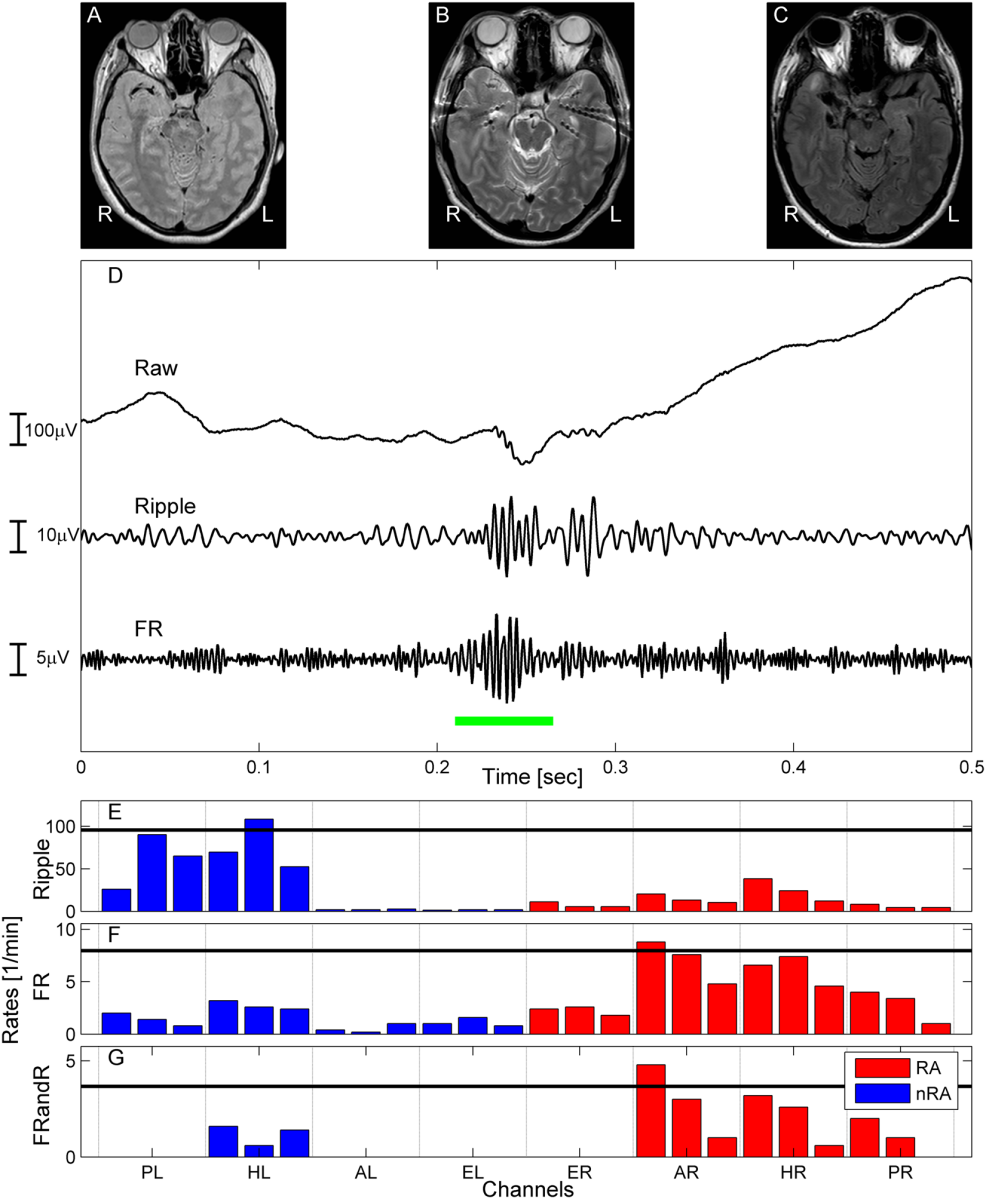
Automated HFO analysis in patient 4. (**A**) Presurgical MRI shows right hippocampal sclerosis (HS). (**B**) MRI after the implantation of 8 depth electrodes sampling the mesial temporal structures. (**C**) MRI after right selective amygdala-hippocampectomy, resulting in long-term seizure freedom. (**D**) Example of a ripple co-occurring with a FR (FRandR, green line). (**E**,**F**,**G**) The HFO types differ in their distribution over channels. Given the neuroradiological diagnosis of this patient, we present here the electrode contacts located in the mesial temporal structures. Note that several channels have FRandR rate = 0. Channels with rates above the 95 percentile (black horizontal line) were taken to define the HFO area. Channels inside the resected area (RA) are shown in red, channels outside the RA in blue. The ripple area was not resected (**E**). The resection of the FRandR area (**G**, channel AR1-AR2) led to seizure freedom. Implantation sites: AL amygdala left; AR amygdala right; EL entorhinal cortex left; ER entorhinal cortex right; HL anterior hippocampus left; HR anterior hippocampus right; PL posterior hippocampus left; PR posterior hippocampus left.

**Figure 2:**
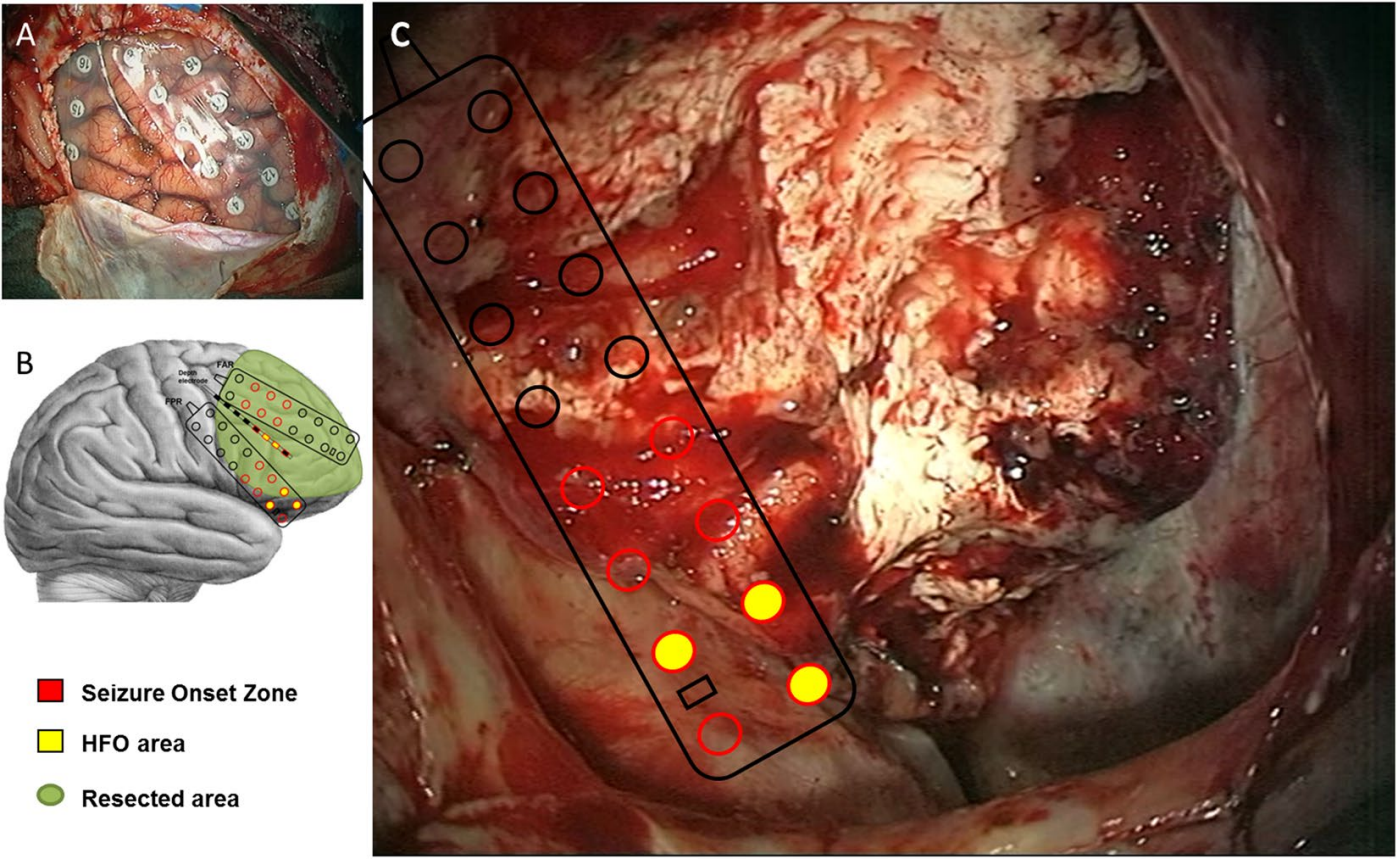
The HFO area exceeds the resected area in the patient with recurrent seizures (patient 17). (**A**) Coverage of the implanted electrodes. (**B**) Reconstruction of surgical resection. Red electrode contacts denote the seizure onset zone (SOZ). The HFO area (contacts filled yellow) is smaller than the SOZ. (**C**) The resected area (RA) does not comprise all channels of the HFO area. The patient suffers from recurrent seizures. Full resection of the HFO area might have achieved seizure freedom.

Automatic HFO detection yielded ripples (median amplitude 27.4 µVpp (peak-to-peak), interquartile range 15.0 µVpp) and FR (median  amplitude 9.2 µVpp, interquartile range 7.5 µVpp). For each individual patient, we determined whether the HFO area was fully or partly resected and whether seizure freedom was achieved (Table S1). The mean follow-up for poor outcome (29 ± 15 mo) was longer than for good outcome (23 ± 11 mo) but the two distributions are indistinguishable (Wilcoxon rank sum test, p = 0.25). When defining the HFO area by FRandR (FRandR area, Fig. 1), we achieved specificity 100%, CI [78–100%], which was more specific in predicting seizure freedom than FR and ripples separately (Table 2). The high specificity indicates that our algorithm provides results consistent with the current surgical planning. The low sensitivity (57%, CI [18–90%]) is due to the small number of TP cases and to the large number of FN cases. Both in our analysis and current surgical planning, FN cases may stem from the limited coverage of the implanted electrodes in FN. Analogous considerations hold for the NPV (81%, CI [54–96%]). When taking as outcome measure the Engel class I (ILAE 1–3), values improve to sensitivity 80%, CI [28–99%], NPV 94%, CI [70 100%], and accuracy 95%, CI [75 100%], p = 0.001 (chi2-test). Still, the sensitivity = 57% and the PPV = 100% indicate that the algorithm might have improved surgical planning in the four TP patients in whom the FRandR area was not fully resected and who suffer from recurrent seizures (Fig. 2). Therefore, the overall accuracy of our algorithm (85%, CI [62–97%], p = 0.002 chi2-test) compares well with the seizure-free rate achieved by current surgical planning (65%, CI [41–85%]).

**Table 2:** HFO area and seizure outcome.

	Ripple	FR	FRandR
**Specificity [%]** specificity = TN/(TN + FP)	**54**	**69**	**100**
**Sensitivity [%]** sensitivity = TP/(TP + FN)	**43**	**29**	**57**
**Negative Predictive Value NPV [%]** NPV = TN/(TN + FN)	**64**	**64**	**81**
**Positive Predictive Value PPV [%]** PPV = TP/(TP + FP)	**33**	**33**	**100**
**Accuracy [%]** accuracy = (TP + TN)/N	**50**	**55**	**85**

Defining the HFO area by FR co-occurring with ripples was more specific in predicting seizure freedom than FR and ripples separately. False Negative cases may reflect the limited coverage of the implanted electrodes. Abbreviations: FN = False Negative; FP = False Positive; FR = fast ripple; FRandR = FR co-occurring with ripple; NPV = Negative Predictive Value; PPV = Positive Predictive Value; TN = True Negative; TP = True Positive; N = number of patients.

### Test-retest reliability of the spatial distribution of HFO rates

**Figure 3:**
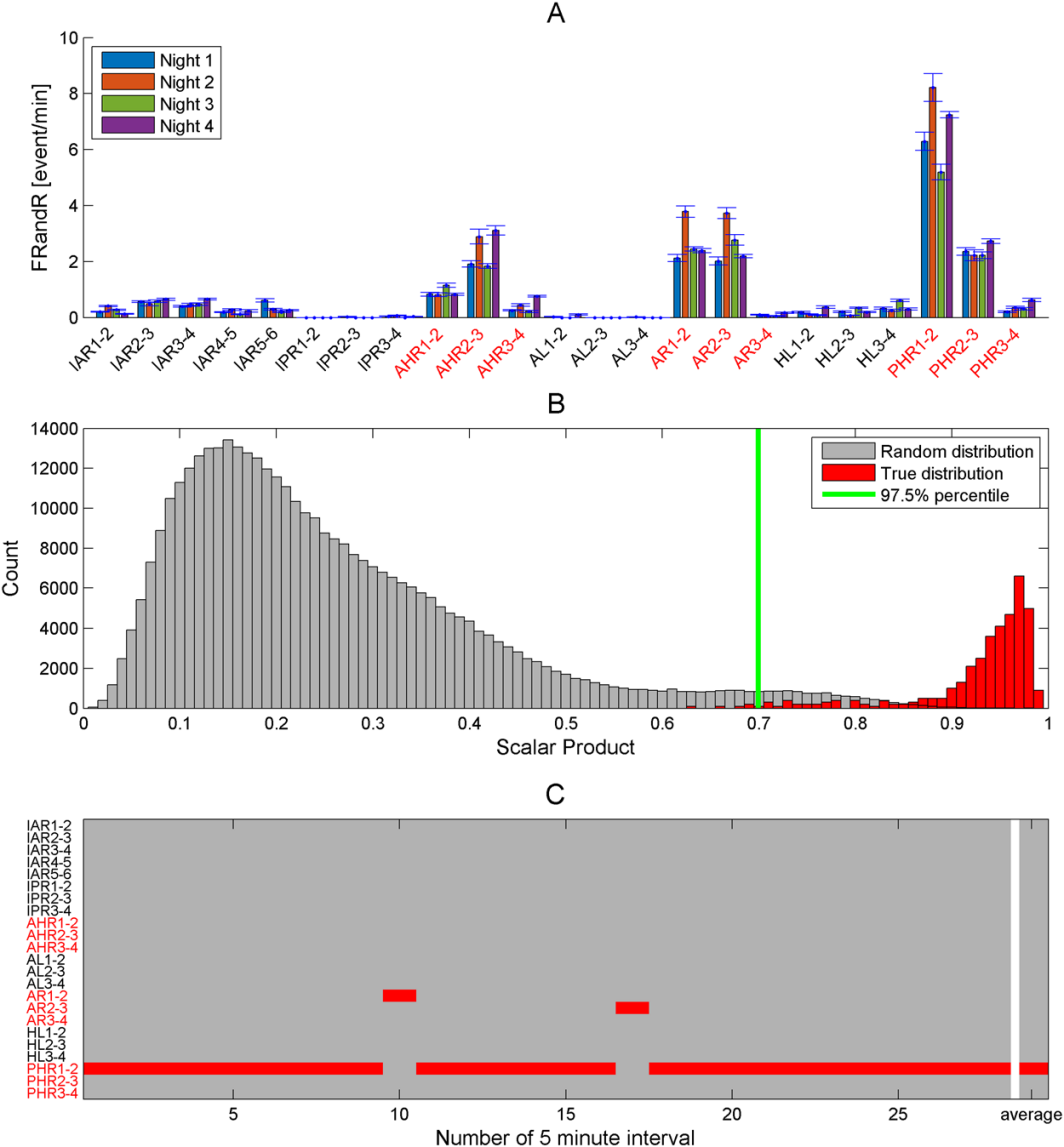
Test-retest analysis of FRandR rates in patient 1. Names of resected channels are highlighted in red. The patient achieved seizure freedom. (**A**) Rates of FRandR (co-occurring ripple and fast ripple, events/min) from 4 nights are plotted. Variability across intervals within nights is indicated by standard error bars. The HFO area comprises channel PHR1-2 for each night. The HFO area was stable over nights and included in the RA. (**B**) Reproducibility of the HFO area. The true distribution of the normalized scalar product of HFO rates for each pair of intervals is plotted in red. The random distribution of the normalized scalar product of HFO rates obtained by permutation analysis is plotted in gray (5000 permutations). The 97.5 percentile of the random permutation is indicated in green. 99% of the true distribution exceeded significance, which indicates a strong stability of the HFO area over time. (**C**) FRandR area for all intervals. The HFO area is determined for each interval by rates thresholding and the corresponding channels are marked in red. The HFO area was inside the resected area for all intervals. The HFO area averaged over all intervals was included in the resection area, which correctly predicted seizure freedom.

To evaluate the reproducibility of the spatial distribution of HFO, we calculated the HFO rates during several periods of slow wave sleep for 18 patients. To illustrate the procedure, the test-retest HFO analysis for Patient 1 is presented in Fig. 3. First, we quantified the HFO rates for different nights (Fig. 3A). We then computed the scalar product for all pairs of HFO vectors within and between nights and tested significance against a random distribution (Fig. 3B).

Across the group of 18 patients (Table 1), the percentage of significant scalar products had median of 97.5% (lower quartile 67%) when taking pairs of all 5 minutes intervals, and a median of 100% (lower quartile 100%) when taking pairs of entire nights. Over the intervals of TLE patients, the median was >98% irrespective of outcome. Over the intervals of ETE patients, the median was 97% for patients with good outcome and only 36% for patients with poor outcome.

### Spectral analysis of ripples co-occurring with FR

**Figure 4:**
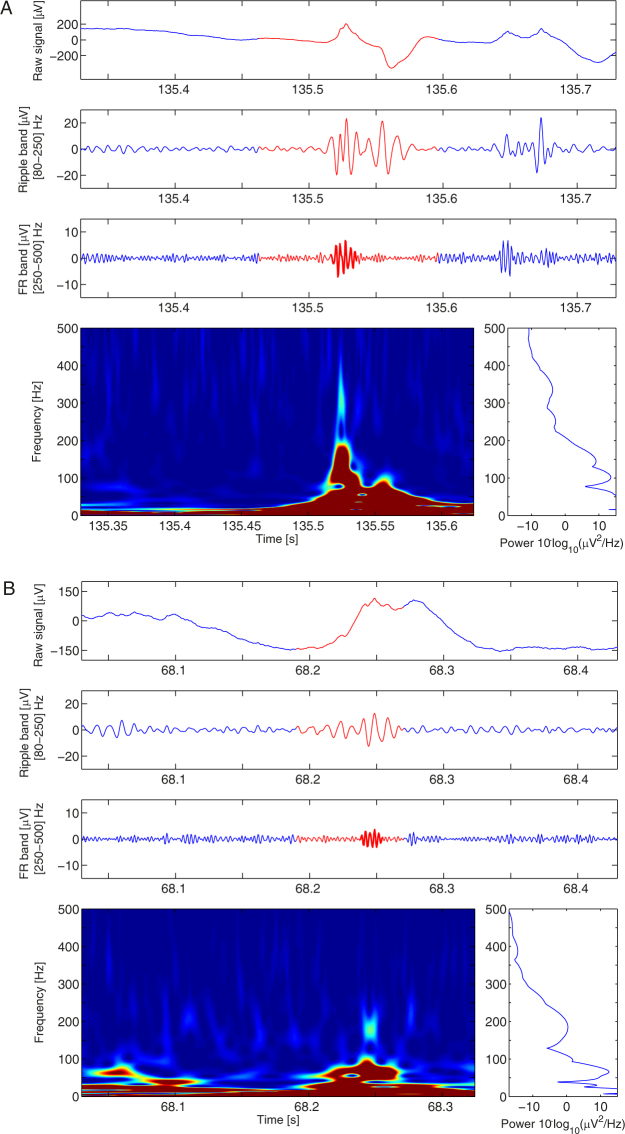
Spectral analysis of FRandR events. We provide here two examples of FRandR events with bimodal (**A**) and unimodal (**B**) frequency spectrum, respectively. From top to bottom: the raw data, the signal filtered in the ripple band (80–250 Hz), the signal filtered in the FR band (250–500 Hz), the Stockwell time-frequency transform, the instantaneous power spectrum averaged over the duration of the FR. The duration of the FRandR is highlighted in red, the FR in bold. (**A**) A blob appears in the time-frequency domain at latency 135.53 s and central frequency 350 Hz in the FR spectral range. This explains the event in the FR band filtered signal. (**B**) A blob appears in the time-frequency domain at latency 68.25 s and central frequency 190 Hz in the ripple spectral range. Energy of the broad peak in the ripple band spreads to higher frequencies and appears as an event in the FR band filtered signal.

In order to identify the spectral signature of FRandR, we selected the bipolar channel with highest FRandR rate included in the HFO area of each patient. For each detected FRandR, we computed the instantaneous power spectrum and detected peaks and troughs in the HFO range (80–500 Hz). Out of 6182 detected events, 45% exhibited two distinct peaks in ripple and FR band with the second peak at median frequency 240 Hz (Fig. 4A), 28% had only one peak in the HFO range (median frequency 184 Hz, Fig. 4B), and 27% did not show a prominent peak >100 Hz in the HFO range. No significant difference was observed between TLE and ETE channels. Since the algorithm does not use the power spectrum to detect a single event, the finding of a prominent spectral peak indicates that an HFO is a distinct spectral entity.

## Discussion

Building on a prospective definition of HFO, our algorithm classified tissue sampled by each individual electrode as epileptogenic or normal as validated by the prediction of seizure outcome.

### FRandR predict seizure freedom in the individual patient

As a main strength of our study, the resection of FRandR provided a high specificity in predicting seizure freedom (13/13 patients, 100%) with narrow confidence intervals (75–100%). Such high specificity not only generalizes the value of FRandR across different types of patients, but still holds true at the level of the individual patient. Different from other studies that consider only HFO summed across the SOZ^13,23^, we based our analysis on post-surgical seizure freedom, which is a prerequisite to guide epilepsy surgery.

In all seizure free patients with ETE (patients 10–16) the RA was limited to 1–2 cm^2^ whereas the SOZ extended also to eloquent cortex. While the SOZ was therefore not fully resected in all ETE patients, the HFO area as defined by FRandR was always fully resected. Thus, compared to the SOZ, FRandR was a more specific marker of the EZ in the invidual patient.

### FRandR in patients with recurrent seizures

Among the 7 patients with recurrent seizures, FRandR were fully resected in 3 FN and not fully resected in 4 TP, resulting in a sensitivity of 57% CI [18–90%]. Among the 3 FN (15%), in patient 9 the HFO area was fully included in the RA but seizures recurred, which may reflect the limited coverage of the implanted electrodes. Patients 7 and 8 were seizure free under antiepileptic drugs but missed the intake of one dosage in noncompliance and consequently suffered a single recurrent seizure, so their seizure outcome was rated ILAE 3. In these 3 cases, HFO guidance was at least consistent with routine surgical planning.

There were 4 patients where the HFO area was not fully resected and who suffered from recurrent seizures (TP). In patient 17 the RA was much larger than the 1–2 cm^2^ mentioned above and almost the entire right frontal cortex was resected. While parts of the HFO area were indeed included in this resection, the resection of the HFO area was not complete. (Fig. 2). In patient 18, our analysis identified the HFO area over occipital contacts ipsilateral to the resection, which included parietal cortex. Patient 19 was implanted with a parietal 4 × 8 electrode grid and a temporal 2 × 8 temporal grid covering multiple cavernoma. The HFO area included the parietal cavernoma but not the temporal cavernoma. The surgical choice was to resect only the temporal cavernoma. In patient 20, the resection included the seizure onset area in the posterior margin of the left occipito-temporal gyrus but the HFO area was in the anterior margin. In these patients, HFO guidance may have improved the routine surgical planning that defined the RA.

### Automated detection predicts seizure outcome

Our automated analysis provides a prospective definition of the HFO area, which was then subjected to an unbiased evaluation of its clinical relevance. In contrast, expert observers’ HFO analysis suffers from time-consuming visual marking and lacks reproducibility^13,23,24^.

In our study, the high reproducibility of the spatial HFO distribution within and between nights confirms the clinical value of HFO as a reliable biomarker for epileptogenic tissue. Interestingly, ETE patients with poor outcome stood out in the low reproducibility of their HFO distribution. The test-retest analysis thus not only shows the reliability of the HFO analysis, but it may even provide a level of confidence for its diagnostic power.

The most rigorous validation for HFO analysis is its comparison with post-surgical outcome. To date, only a few studies correlated automated HFO analysis with patients’ outcome^21,22^. Our algorithm used here was trained and tested on datasets from different epilepsy centres and marked by different expert viewers^22^. As a major advance over previous studies, we applied here a fully automated procedure. With minimal clinical information and minimal human monitoring of data quality, our automated algorithm analysed the iEEG by detecting HFOs and by rate thresholding to delineate the HFO area, which was then validated against clinical outcome.

### Limitations of rate thresholding

In principle, rate thresholding could compromise the HFO area as a guide to the EZ. For example, if the EZ were not sampled by the electrodes at all, the HFO area would appear at a spurious location outside the EZ. Given the quality of our surgical planning, this did not occur in our patient series. As another limitation, the size of the HFO area will depend on the proportion of electrodes covering the EZ. This was, however, not a relevant source of error in our patient series, where the HFO area always provided an estimate of the EZ that seemed reasonable to the surgeon. It has to be kept in mind that HFO analysis is not intended to replace routine surgical planning and overrule the surgeon’s decision but that it might provide added value in ambiguous cases.

### FRandR are more specific than FR and ripple separately

The frequency bands for ripple (80–250 Hz) and FR (250–500 Hz) are often used in the literature^23^ and we therefore designed our algorithm to detect HFO patterns separately in these two bands. FRs are considered more specific for epileptogenicity than ripples because of their close relation to the SOZ^25^ and to the seizure outcome^10,12^. Surprisingly, the co-occurrence of FRs and ripples was not evaluated before. Here we demonstrated that the resection of FRandR is more specifically associated with seizure freedom than the resection of ripples and FRs separately.

In the instantaneous power spectrum, FRandR had at least one prominent peak around 200 Hz (median bimodal FRandR: 240 Hz, median unimodal FRandR: 184 Hz). In the high-pass filtered data (>250 Hz) the contribution of this peak matched the criteria for FR detection. In about half of the detected events (bimodal), there was a simultaneous occurrence of a ripple peak and a FR peak, suggesting the presence of two separate spectral entities. In the other half of the detected events (unimodal), a major spectral contribution in the ripple band spread over to the FR band. Our recording setup and our prospective definition of HFO includes both bimodal and unimodal events for the prediction of surgery outcome.

It remains open whether ripples and FR (or very high frequency oscillations^26^) reflect distinct biological processes. HFO, and in particular FR, are thought to express abnormal connectivity patterns reflecting a local imbalance between excitatory and inhibitory circuitry in the epileptogenic zone^27^. One direction of further investigation of the generative processes in humans might be the combination of macro- and micro-electrode recordings^28–30^. Such a combination of electrodes probing different spatial extent might shed light on the complex spatio-temporal synchronization patterns and neuronal networks that generate HFO.

### Generalizability

The results of our algorithm suggest that the information provided by prospectively defined HFOs could contribute to surgery planning in cases where the extent of surgical resection can be adapted. As an example, the exact extent of focal cortical dysplasia (FCD) is sometimes challenging to detect in MRI^31^ and remains difficult to delineate^32^. It is in these patients where complementary electrophysiological markers such as HFO may be useful^9,12,33^.

In general, several methods are currently discussed to improve surgery outcome. Promising are, e.g. strategies based on patient’s clinical characteristics^34,35^. While HFO are spatio-temporally highly localized, the analysis of functional connectivity between electrode sites aims to identify the nodes with higher seizure likelihood, both during surgery^36,37^ and during the preoperative period^38,39^. Given the two different spatio-temporal scales in iEEG analysis, the two approaches appear complementary and their combination might add to the understanding of the underlying pathophysiology.

In our study, the resection of the prospectively defined HFO area proved to be highly specific and reproducible in all patients with seizure freedom, while it may have improved the outcome in four patients with recurrent seizures. The HFO area was defined by a fully automated algorithm. We thus validated the HFO area against outcome in the individual patient, which is a prerequisite before HFOs can guide surgical treatment in multicentre studies.

## Methods

### Patient selection

We included all consecutive patients with drug-resistant focal epilepsy in our centre that 1) underwent invasive EEG recordings with subdural and/or depth electrodes from March 2012 to April 2016 as part of their presurgical evaluation, 2) consequently underwent resective surgery, and 3) were followed-up for >1 year after surgery in case of seizure freedom. The postsurgical seizure outcome was determined in follow-up visits and classified according to the International League Against Epilepsy (ILAE)^40^.

### Ethics statement

Collection of personal patient data and retrospective scientific workup was approved by the research ethics committee (Kantonale Ethikkommission KEK-ZH-Nr. 2012-0212) and collection of patients’ written informed consent was waived. The study was performed in accordance with the relevant guidelines and regulations.

### Electrode types and implantation sites

Subdural strip and grid electrodes as well as depth electrodes were placed according to the findings of the non-invasive presurgical evaluation (Fig. 1A,B).

In TLE patients, depth electrodes (1.3 mm diameter, 8 contacts of 1.6 mm length, spacing between contacts centres 5 mm, ADTech®, www.adtechmedical.com) were implanted stereotactically into the amygdala, the hippocampal head and the entorhinal and perirhinal cortex bilaterally.

In ETE patients, a combination of depth and subdural grid and strip electrodes (contact diameter 4 mm with a 2.3 mm exposure, spacing between contact centers 10 mm, ADTech®) was placed after craniotomy. Post-implantation MR images were used to locate each contact anatomically along the electrode trajectory.

### Surgical planning

The decision for resection surgery was based on non-invasive investigations as well as on intracranial investigations^1^. The results of HFO analysis were not used for surgical planning.

### Data acquisition

Intracranial data was acquired at 4000 Hz sampling frequency with an ATLAS recording system (0.5–1000 Hz pass-band, Neuralynx, www.neuralynx.com) and down-sampled to 2000 Hz for HFO analysis. In addition, scalp EEG according to the 10–20 system, with minor adaptations in order to avoid the surgical scalp lesions, and the submental electromyogram (EMG) were recorded. The iEEG was recorded against a common intracranial reference and then transformed to bipolar channels for further analysis.

### Data selection

From each night recording we selected up to six intervals of five minutes of interictal slow-wave sleep that promotes low muscle activity and high HFO rates^14,41^. Sleep scoring was performed based on scalp EEG, electro-oculogram, EMG and video recordings. We chose time intervals at least three hours apart from epileptic seizures in order to eliminate the influence of seizure activity on our analysis. We excluded all electrode contacts where electrical stimulation evoked motor or language responses (Table S1). In TLE patients, we included only the 3 most mesial bipolar channels. The amount of nights and intervals varied across patients (Table 1).

### Prospective definition of HFOs

HFOs were defined prospectively by an automated detector, which we had previously trained, tested and validated to detect visually marked events in datasets from the Montreal Neurological Institute^22^.

The detector incorporates information from both time and frequency domain and operates in two stages. In the first stage – baseline detection – baseline segments with low oscillatory activity were identified by the Stockwell entropy distribution^17,42^. The distribution of the envelope of baseline segments was used to define an amplitude threshold^22^. The second stage – HFO detection – was conducted separately for ripples (band-pass 80–240 Hz, stopband 70 Hz and 250 Hz, FIR equiripple filter with stopband attenuation 60 dB) and FRs (band-pass 250–490 Hz, stopband 240 Hz and 500 Hz). Events with the envelope of the filtered signal exceeding the amplitude threshold for at least 20 ms/10 ms were labelled as ripples/FR (Fig. 1D). The algorithm then identified FRs overlapping with a ripple, which we defined as a third type of HFO: FR co-occurring with ripples (FRandR). As an advantage over other automatic detectors, this algorithm reports several channels where no HFO occur (Fig. 1G).

The code is freely available at the HFO detectors repository on github (https://github.com/HFO-detect/HFO-detect-matlab). The iEEG data with the markings of our HFO events is freely available at CRCNS.org http://dx.doi.org/10.6080/K06Q1VD5.

### Definition of the HFO area by rate thresholding

We analysed the spatial distribution of HFO rates across channels in each patient. The ensemble of those channels whose rates exceeded the rate threshold (95 percentile of the HFO rate distribution) was defined as the HFO area (Fig. 1E–G). The 95 percentile was computed by the Matlab function prctile.m. To compare HFO types, we identified an HFO area for all three types of HFOs (ripples, FRs and FRandR).

### Test-retest reliability of the spatial distribution of HFO

We quantified the the test-retest reliability of the distribution of HFO rates over the ensemble of slow wave sleep 5 minutes intervals (Fig. 3A). For each interval pair we computed the normalized scalar product of the spatial distribution of the HFO rates. The scalar product is ~1 for highly overlapping spatial distributions of HFO rate, and lower otherwise. To test the magnitude of the true scalar product against chance, we constructed a distribution of scalar products by randomly permuting (N = 5000) the order of channels for each interval. The true value of the scalar product was considered statistically significant if it exceeded the 97.5% percentile of the distribution^43^ (Fig. 3B). Next, we took the mean HFO rate for each night and tested reproducibility over nights (Fig. 3C).

### Clinical validation of HFO against seizure outcome

We evaluated whether the HFO area was included in the resection area to quantify the predictive value of the HFO area with respect to seizure outcome (Table 1). Automated HFO detection and analysis were blind to clinical information. Following^12^, we defined as true positive (TP) a patient where the HFO area was not fully located within the RA, i.e. at least one channel of the HFO area was not resected, and the patient suffered from recurrent seizures (ILAE 2–6). We defined as false positive (FP) a patient where the HFO area was not fully located inside the RA but who achieved seizure freedom (ILAE 1). We defined as false negative (FN) a patient where the HFO area was fully located within the RA but who suffered from recurrent seizures. We defined as true negative (TN) a patient where the HFO area was fully located inside the RA and who became seizure free. The positive predictive value was calculated as PPV = TP/(TP + FP), negative predictive value as NPV = TN/(TN + FN), sensitivity = TP/(TP + FN), specificity = TN/(TN + FP), and accuracy = (TP + TN)/N. Estimates of the 95% confidence intervals (CI) used the binomial method for sample size <10 and the asymptotic method for sample size >10 based on the normal approximation. Statistical significance was established at p < 0.05.

### Spectral analysis of ripples co-occurring with FR

For each patient we selected the bipolar channel with highest FRandR rate included in the HFO area. We computed the Stockwell transform of the signal in an interval of 0.6 s around each detected FRandR. We extracted the averaged instantaneous spectrum over the duration of the detected FR and detected peaks and troughs in the HFO frequency band [80 500] Hz after least-squares fitting maximum likelihood estimation with gaussian distribution (four gaussians, lsqcurvefit.m in Matlab). We defined bimodal events as events with a trough between 150 and 250 Hz, and at least one peak above 200 Hz. We defined unimodal events as events with a single peak >100 Hz that is detectable in the ripple band.

## Electronic supplementary material

Supplementary Information
