# Supplementary material for: Resection of high frequency oscillations predicts seizure outcome in the individual patient
Source: Sci Rep. 2017 Oct 23;7:13836. doi: 10.1038/s41598-017-13064-1 (PMC5653833; doi:10.1038/s41598-017-13064-1)
Supplement: Supplementary file 1 — Supplementary Information [file 41598_2017_13064_MOESM1_ESM.pdf]

Resection of high frequency oscillations predicts seizure outcome in the individual patient

Tommaso Fedele, PhD[1,*]; Sergey Burnos, MSc[1,2,*]; Ece Boran, MSc[1]; Niklaus Krayenbühl, MD[1]; Peter Hilfiker, PhD[3]; Thomas Grunwald, PhD, MD[3]; Johannes Sarnthein, PhD[1,4]

[1] University Hospital Zurich, Neurosurgery Department, Zurich, Switzerland

[2] ETH Zurich, Institute of Neuroinformatics, Zurich, Switzerland

[3] Swiss Epilepsy Centre, Zurich, Switzerland

[4] University of Zurich, Zurich Neuroscience Centre, Zurich, Switzerland

* These authors contributed equally to the manuscript

Corresponding author: Tommaso Fedele

Klinik für Neurochirurgie, UniversitätsSpital Zürich,

8091 Zürich, Switzerland

Tel: +41 44 255 5672

tommaso.fedele@usz.ch

SUPPLEMENTARY MATERIAL

Table 1S: HFO rates of all patients. For each patient, we list the postsurgical seizure outcome (ILAE) and the names of the implanted channels. Channels excluded from the analysis are marked in gray. The resected area (RA) is highlighted in red. The rates of ripple, FR and FRandR events are given in events/minute for each channel. Channels with the HFO rate exceeding the 95% percentile of the HFO distribution define the HFO area and are highlighted in green.

Patient 1 ILAE 1

Channels	'IAR1-2'	'IAR2-3'	'IAR3-4'	'IAR4-5'	'IAR5-6'	'IPR1-2'	'IPR2-3'	'IPR3-4'	'AHR1-2'	'AHR2-3'	'AHR3-4'	'AL1-2'	'AL2-3'	'AL3-4'	'AR1-2'	'AR2-3'	'AR3-4'	'HL1-2'	'HL2-3'	'HL3-4'	'PHR1-2'	'PHR2-3'	'PHR3-4'
Ripples	33.8	55.2	40.2	26.7	31.1	0.6	1.7	11.8	19.8	21.4	19.7	3.2	0.6	0.2	47.8	48.2	4.3	49.3	60.6	105.1	22.8	20.2	11.8
FR	2.9	3.2	2.9	2.8	2.8	2.4	2.6	2.4	3.3	4.8	3.0	2.3	2.7	2.6	5.3	5.1	2.8	2.5	2.2	2.5	10.2	4.9	3.0
FRandR	0.3	0.6	0.5	0.2	0.4	0.0	0.0	0.0	0.9	2.4	0.4	0.0	0.0	0.0	2.7	2.7	0.1	0.2	0.2	0.4	6.7	2.4	0.4

Patient 2 ILAE 1

Channels	'AL1-2'	'AL2-3'	'AL3-4'	'AR1-2'	'AR2-3'	'AR3-4'	'EL1-2'	'EL2-3'	'EL3-4'	'ER1-2'	'ER2-3'	'ER3-4'	'HL1-2'	'HL2-3'	'HL3-4'	'HR1-2'	'HR2-3'	'HR3-4'	'PL1-2'	'PL2-3'	'PL3-4'	'PR1-2'	'PR2-3'	'PR3-4'
Ripples	44.3	17.7	5.1	52.6	16.2	1.4	2.6	1.5	2.7	51.4	11.7	0.6	4.8	1.8	1.8	71.4	19.7	1.6	16.3	2.8	0.4	48.9	18.1	0.8
FR	9.1	4.2	3.4	16.3	3.5	1.6	2.0	2.4	1.8	5.4	2.6	1.7	2.7	1.9	2.2	14.1	2.6	2.6	13.1	6.0	2.5	16.2	6.9	2.5
FRandR	4.6	1.4	0.9	7.5	1.0	0.0	0.0	0.0	0.1	2.7	0.2	0.0	0.8	0.0	0.0	6.7	0.3	0.0	1.7	0.2	0.1	9.8	3.7	0.0

Patient 3 ILAE 1

Channels	'AHL1-2'	'AHL2-3'	'AHL3-4'	'AHR1-2'	'AHR2-3'	'AHR3-4'	'AL1-2'	'AL2-3'	'AL3-4'	'ECL1-2'	'ECL2-3'	'ECL3-4'	'PHL1-2'	'PHL2-3'	'PHL3-4'
Ripples	20.2	7.0	2.1	211.9	159.0	233.8	1.6	0.7	0.2	2.0	0.4	0.1	8.0	1.0	0.7
FR	15.5	7.3	2.5	10.1	5.8	5.1	2.4	2.3	2.5	2.7	2.7	2.8	3.5	3.0	6.8
FRandR	9.1	2.6	0.0	5.2	2.4	2.5	0.0	0.0	0.0	0.0	0.0	0.0	1.2	0.4	0.1

Patient 4 ILAE 1

Channels	'AL1-2'	'AL2-3'	'AL3-4'	'AR1-2'	'AR2-3'	'AR3-4'	'EL1-2'	'EL2-3'	'EL3-4'	'ER1-2'	'ER2-3'	'ER3-4'	'HL1-2'	'HL2-3'	'HL3-4'	'HR1-2'	'HR2-3'	'HR3-4'	'PL1-2'	'PL2-3'	'PL3-4'	'PR1-2'	'PR2-3'	'PR3-4'
Ripples	1.0	0.9	0.2	39.6	32.5	11.0	0.3	0.1	0.1	3.9	1.7	3.0	140.6	128.2	63.8	41.6	36.5	20.4	82.6	160.3	44.6	12.2	3.7	0.6
FR	2.3	2.3	2.8	23.4	18.0	5.5	2.8	2.9	2.4	2.9	2.9	2.5	5.7	5.3	4.2	19.0	19.0	13.5	2.5	2.9	2.4	8.4	3.7	2.7
FRandR	0.0	0.0	0.0	14.0	12.3	2.3	0.0	0.0	0.0	0.0	0.0	0.0	1.7	1.6	1.7	9.0	6.4	5.0	0.6	1.2	0.2	5.2	0.6	0.0

Patient 5 ILAE 1

Channels	'AL1-2'	'AL2-3'	'AL3-4'	'AR1-2'	'AR2-3'	'AR3-4'	'EL1-2'	'EL2-3'	'EL3-4'	'ER1-2'	'ER2-3'	'ER3-4'	'HL1-2'	'HL2-3'	'HL3-4'	'HR1-2'	'HR2-3'	'HR3-4'	'PL1-2'	'PL2-3'	'PL3-4'	'PR1-2'	'PR2-3'	'PR3-4'
Ripples	14.2	3.3	1.8	43.0	30.1	8.2	2.8	2.6	1.1	6.9	4.7	6.6	4.0	3.3	1.4	18.4	7.2	1.6	17.6	3.3	1.6	117.1	25.3	5.8
FR	3.8	2.8	2.4	12.6	2.6	2.4	2.6	2.4	2.1	3.2	2.5	2.5	2.3	2.6	2.2	3.1	2.4	2.3	2.7	2.5	2.0	14.0	3.4	2.0
FRandR	0.7	0.3	0.0	6.2	0.3	0.1	0.3	0.1	0.0	0.4	0.1	0.1	0.2	0.2	0.0	0.4	0.0	0.0	0.1	0.1	0.0	9.4	0.7	0.1

Patient 6 ILAE 1

Channels	'AL1-2'	'AL2-3'	'AL3-4'	'AR1-2'	'AR2-3'	'AR3-4'	'EL1-2'	'EL2-3'	'EL3-4'	'ER1-2'	'ER2-3'	'ER3-4'	'HL1-2'	'HL2-3'	'HL3-4'	'HR1-2'	'HR2-3'	'HR3-4'	'PL1-2'	'PL2-3'	'PL3-4'	'PR1-2'	'PR2-3'	'PR3-4'
Ripples	83.8	55.9	5.3	27.0	20.3	2.8	2.3	1.5	0.6	13.6	9.6	0.7	0.1	0.1	0.0	0.4	0.3	0.2	45.4	76.3	1.9	147.3	14.2	0.5
FR	9.2	5.5	2.6	8.6	6.2	2.7	2.3	2.6	2.3	3.6	3.0	2.2	2.3	2.2	2.6	2.4	2.5	2.7	2.4	2.2	2.4	18.1	11.6	2.7
FRandR	4.3	2.4	0.0	4.0	3.2	0.1	0.0	0.0	0.0	0.9	0.5	0.0	0.0	0.0	0.0	0.0	0.0	0.0	0.1	0.4	0.0	7.6	2.8	0.2

Patient 7 ILAE 3

Channels	'AR1-2'	'AR2-3'	'AR3-4'	'AL1-2'	'AL2-3'	'AL3-4'	'HR1-2'	'HR2-3'	'HR3-4'	'HL1-2'	'HL2-3'	'HL3-4'	'ER1-2'	'ER2-3'	'ER3-4'	'EL1-2'	'EL2-3'	'EL3-4'	'PR1-2'	'PR2-3'	'PR3-4'	'PL1-2'	'PL2-3'	'PL3-4'
Ripples	4.4	3.0	2.6	11.0	3.8	0.6	42.6	69.0	75.6	15.8	4.8	1.2	1.0	2.4	2.4	22.4	1.4	6.2	11.8	23.8	20.6	72.2	57.6	3.6
FR	5.2	3.4	0.6	5.6	3.4	3.6	17.4	21.8	27.0	36.2	18.0	6.0	3.4	3.8	5.2	5.0	2.0	3.2	6.0	6.2	4.6	7.8	17.4	5.0
FRandR	0.4	0.0	0.0	1.0	0.0	0.0	6.0	7.6	7.6	7.4	1.6	0.0	0.0	0.0	0.0	0.6	0.0	0.0	2.0	2.2	1.4	4.0	8.2	0.2

Patient 8 ILAE 3

Channels	'AL1-2'	'AL2-3'	'AL3-4'	'AR1-2'	'AR2-3'	'AR3-4'	'EL1-2'	'EL2-3'	'EL3-4'	'ER1-2'	'ER2-3'	'ER3-4'	'HL1-2'	'HL2-3'	'HL3-4'	'HR1-2'	'HR2-3'	'HR3-4'	'PL1-2'	'PL2-3'	'PL3-4'	'PR1-2'	'PR2-3'	'PR3-4'
Ripples	18.4	1.9	0.1	4.6	6.2	1.7	3.4	1.1	0.3	2.6	2.8	2.6	4.1	1.7	0.5	3.9	4.4	0.8	2.5	0.1	0.1	1.8	0.3	0.2
FR	11.5	3.1	1.9	2.9	2.2	2.4	5.9	2.2	2.4	4.8	3.5	2.2	2.3	2.4	2.1	4.7	5.2	2.8	3.8	1.7	2.1	3.8	2.2	2.3
FRandR	7.4	0.0	0.0	0.1	0.1	0.0	0.8	0.0	0.0	1.1	0.6	0.1	0.0	0.0	0.0	1.5	1.7	0.2	0.5	0.0	0.0	0.7	0.1	0.0

Patient 9 ILAE 5

Channels	'AL1-2'	'AL2-3'	'AL3-4'	'AR1-2'	'AR2-3'	'AR3-4'	'EL1-2'	'EL2-3'	'EL3-4'	'ER1-2'	'ER2-3'	'ER3-4'	'HL1-2'	'HL2-3'	'HL3-4'	'HR1-2'	'HR2-3'	'HR3-4'	'PL1-2'	'PL2-3'	'PL3-4'	'PR1-2'	'PR2-3'	'PR3-4'
Ripples	35.7	12.8	3.6	22.9	7.3	1.6	59.0	27.2	1.9	22.0	9.9	6.8	64.7	14.4	2.9	23.3	23.3	15.8	28.3	14.5	6.6	52.5	6.7	4.9
FR	11.5	5.5	5.3	5.5	2.2	2.5	4.5	2.7	2.0	5.8	1.9	2.3	42.5	2.2	1.9	8.5	2.6	2.3	11.1	5.5	3.2	6.2	2.0	2.0
FRandR	7.0	0.8	1.0	2.6	0.1	0.0	1.5	0.2	0.0	1.1	0.1	0.0	27.5	0.2	0.0	4.0	0.8	0.1	5.9	1.2	0.2	3.6	0.2	0.1

Patient 10 ILAE 1

Channels	'GR1-2'	'GR2-3'	'GR3-4'	'GR4-5'	'GR5-6'	'GR6-7'	'GR7-8'	'GR9-10'	'GR10-11'	'GR11-12'	'GR12-13'	'GR13-14'	'GR14-15'	'GR15-16'	'GR17-18'	'GR18-19'	'GR19-20'	'GR20-21'	'GR21-22'	'GR22-23'	'GR23-24'	'GR25-26'	'GR26-27'	'GR27-28'
Ripples	9	14	11	4	2	29	57	42	44	60	38	25	35	61	5	13	9	3	8	14	96	0	2	3
FR	3	1	2	2	2	3	3	3	2	2	3	3	3	4	3	3	2	2	2	3	3	3	3	2
FRandR	0	0	0	0	0	0	0	0	0	0	0	0	0	1	0	0	0	0	0	0	1	0	0	0

Channels	'GR28-29'	'GR29-30'	'GR30-31'	'GR31-32'	'IAR1-2'	'IAR2-3'	'IAR3-4'	'IPR1-2'	'IPR2-3'	'IPR3-4'
Ripples	27	37	31	22	5	5	0	131	94	16
FR	2	2	2	2	2	2	2	12	11	3
FRandR	0	0	0	0	0	0	0	5	4	0

Patient 11 ILAE 1

Channels	'GR1-2'	'GR2-3'	'GR3-4'	'GR4-5'	'GR5-6'	'GR6-7'	'GR7-8'	'GR9-10'	'GR10-11'	'GR11-12'	'GR12-13'	'GR13-14'	'GR14-15'	'GR15-16'	'GR17-18'	'GR18-19'	'GR19-20'	'GR20-21'	'GR21-22'	'GR22-23'	'GR23-24'	'GR25-26'	'GR26-27'	'GR27-28'
Ripples	75.1	44.8	65.5	107.3	142.1	84.7	24.0	139.3	144.9	146.6	167.8	145.6	103.4	85.2	106.9	96.2	82.6	136.9	156.7	58.0	84.3	56.2	84.8	70.7
FR	4.6	2.6	2.6	2.8	3.4	3.6	2.2	6.7	6.3	3.9	4.2	3.6	3.4	3.3	5.9	4.6	3.0	3.7	3.7	2.5	3.0	15.5	22.4	10.4
FRandR	1.4	0.4	0.4	0.6	1.0	0.7	0.1	3.0	2.5	1.2	1.4	1.1	0.7	0.5	2.3	1.6	0.4	1.2	1.4	0.3	0.6	4.0	4.7	2.8

Channels	'GR28-29'	'GR29-30'	'GR30-31'	'GR31-32'	'GR33-34'	'GR34-35'	'GR35-36'	'GR36-37'	'GR37-38'	'GR38-39'	'GR39-40'	'GR41-42'	'GR42-43'	'GR43-44'	'GR44-45'	'GR45-46'	'GR46-47'	'GR47-48'	'GR49-50'	'GR50-51'	'GR51-52'	'GR52-53'	'GR53-54'	'GR54-55'
Ripples	42.2	54.8	37.4	28.8	61.3	68.4	66.8	57.5	77.9	48.5	40.7	52.4	45.0	25.9	63.5	114.5	104.9	56.3	68.5	51.7	54.7	5.3	14.4	32.1
FR	4.5	3.6	2.4	2.4	11.3	36.4	34.7	6.6	6.6	2.7	2.4	6.6	9.0	2.6	4.8	5.1	3.6	2.7	5.3	4.2	4.2	2.6	2.4	2.3
FRandR	1.2	0.6	0.2	0.1	2.6	4.1	5.5	1.1	1.5	0.4	0.1	1.7	2.3	0.4	1.2	1.1	0.8	0.3	1.5	0.9	0.9	0.0	0.0	0.1

Channels	'GR55-56'	'GR57-58'	'GR58-59'	'GR59-60'	'GR60-61'	'GR61-62'	'GR62-63'	'GR63-64'	'TR1-2'	'TR2-3'	'TR3-4'	'TR4-5'	'TR5-6'	'TR6-7'	'TR7-8'	'TR8-9'	'TR9-10'	'IAR3-4'	'IAR4-5'	'IAR5-6'	'PLR5-6'	'PLR6-7'	'PLR7-8'	'PLR13-14'
Ripples	38.1	78.5	70.5	38.1	16.6	51.6	87.1	60.2	121.5	109.0	55.1	42.2	23.5	20.6	0.2	0.1	0.2							
FR	2.7	3.3	2.6	2.2	2.0	3.1	3.1	2.4	77.6	60.7	17.6	4.2	3.3	2.7	2.6	2.3	2.7							
FRandR	0.1	0.5	0.3	0.1	0.0	0.4	0.6	0.3	31.4	22.5	6.5	0.9	0.4	0.2	0.0	0.0	0.0							

Channels	'PLR14-15'	'PLR15-16'	'PMR5-6'	'PMR6-7'	'PMR7-8'	'PMR13-14'	'PMR14-15'	'PMR15-16'

Patient 12 ILAE 1

Channels	'GL1-2'	'GL2-3'	'GL3-4'	'GL4-5'	'GL5-6'	'GL6-7'	'GL7-8'	'GL9-10'	'GL10-11'	'GL11-12'	'GL12-13'	'GL13-14'	'GL14-15'	'GL15-16'	'GL17-18'	'GL18-19'	'GL19-20'	'GL20-21'	'GL21-22'	'GL22-23'	'GL23-24'	'GL25-26'	'GL26-27'	'GL27-28'
Ripples	60.9	42.8	53.1	41.1	15.9	53.4	72.6	58.9	61.8	45.0	10.4	38.2	50.3	35.1	61.4	43.5	45.0	52.5	12.6	29.1	37.4	42.8	35.8	36.6
FR	6.7	2.9	2.8	2.7	2.4	2.7	3.5	3.2	3.3	2.8	2.2	2.9	3.0	2.3	3.1	2.7	2.5	3.2	2.7	2.6	2.4	4.0	3.1	3.1
FRandR	0.7	0.2	0.3	0.3	0.0	0.7	1.0	0.3	0.5	0.3	0.0	0.4	0.5	0.1	0.4	0.2	0.2	0.4	0.0	0.3	0.3	0.4	0.3	0.2

Channels	'GL28-29'	'GL29-30'	'GL30-31'	'GL31-32'	'TL1-2'	'TL2-3'	'TL3-4'	'TL4-5'	'TL5-6'	'TL6-7'	'TL7-8'	'TL8-9'	'TL9-10'
Ripples	34.1	40.5	59.1	122.2	19.8	74.9	67.6	36.4	28.7	8.8	2.3	0.1	0.1
FR	2.9	3.3	2.9	3.7	4.1	3.7	2.8	4.2	4.2	2.9	2.1	2.9	2.9
FRandR	0.2	0.2	0.4	1.5	1.7	1.4	0.7	0.9	0.9	0.1	0.0	0.0	0.0

Patient 13 ILAE 1

Channels	'GR1-2'	'GR2-3'	'GR3-4'	'GR9-10'	'GR10-11'	'GR11-12'	'GR17-18'	'GR18-19'	'GR19-20'	'GR25-26'	'GR26-27'	'GR27-28'	'GR28-29'	'GR29-30'	'GR30-31'	'GR31-32'	'GR33-34'	'GR34-35'	'GR35-36'	'GR36-37'	'GR37-38'	'GR38-39'	'GR39-40'	'GR41-42'
Ripples	78.1	54.3	75.8	8.2	25.0	79.9	148.7	158.8	88.3	119.0	136.1	165.1	194.7	210.5	181.8	148.3	121.4	193.4	163.7	141.2	60.2	73.3	80.1	116.2
FR	2.7	2.5	2.9	2.0	2.2	2.4	3.8	4.3	2.7	3.7	3.3	3.7	3.2	6.0	3.7	5.6	3.4	4.0	4.8	3.5	2.4	2.6	3.0	4.6
FRandR	0.6	0.5	0.7	0.0	0.1	0.6	1.6	1.5	0.5	1.1	1.0	1.4	1.4	3.0	1.4	1.7	0.8	1.6	1.9	1.0	0.3	0.4	0.4	1.2

Channels	'GR42-43'	'GR43-44'	'GR44-45'	'GR45-46'	'GR46-47'	'GR47-48'	'GR49-50'	'GR50-51'	'GR51-52'	'GR52-53'	'GR53-54'	'GR54-55'	'GR55-56'	'GR57-58'	'GR58-59'	'GR59-60'	'GR60-61'	'GR61-62'	'GR62-63'	'GR63-64'	'TR1-2'	'TR2-3'	'TR3-4'	'TR4-5'
Ripples	157.4	171.0	197.2	200.8	183.8	130.9	91.1	109.7	157.3	190.2	189.6	173.2	106.1	90.4	110.5	130.5	195.9	169.9	144.9	121.0	23.3	30.9	43.7	29.2
FR	5.3	4.5	5.0	4.1	4.4	3.8	3.6	3.3	4.7	4.9	4.0	2.7	2.6	3.2	4.3	3.7	5.2	5.2	3.5	3.7	9.7	12.4	33.9	10.7
FRandR	1.9	1.7	2.1	1.6	1.8	1.1	0.7	0.7	1.4	2.1	1.4	0.8	0.5	0.7	0.8	1.3	2.3	2.2	1.2	1.3	3.9	4.2	5.0	3.5

Channels	'TR5-6'	'TR6-7'	'TR7-8'	'TR8-9'	'TR9-10'	'GR4-5'	'GR5-6'	'GR6-7'	'GR7-8'	'GR12-13'	'GR13-14'	'GR14-15'	'GR15-16'	'GR20-21'	'GR21-22'	'GR22-23'	'GR23-24'
Ripples	53.8	65.0	47.8	7.4	14.0												
FR	2.7	2.6	2.2	1.9	3.5												
FRandR	0.3	0.5	0.2	0.0	0.6												

Patient 14 ILAE 1

Channels	'IAR1-2'	'IAR2-3'	'IPR1-2'	'IPR2-3'	'IPR3-4'	'PLR1-2'	'PLR2-3'	'PLR3-4'	'PLR4-5'	'PLR9-10'	'PLR10-11'	'PLR11-12'	'PLR12-13'	'PMR1-2'	'PMR2-3'	'PMR3-4'	'PMR4-5'	'PMR9-10'	'PMR10-11'	'PMR11-12'	'PMR12-13'	'TR1-2'	'TR2-3'	'TR3-4'
Ripples	76.0	57.5	88.8	78.8	142.7	17.5	14.8	56.8	80.4	38.6	71.2	64.1	26.0	103.1	98.5	106.4	84.4	92.9	77.5	86.2	85.2	8.1	7.6	8.2
FR	4.3	4.4	2.6	4.9	47.0	3.0	2.4	3.0	3.1	2.7	2.6	3.1	2.5	5.0	5.9	5.6	7.5	5.3	2.6	4.0	6.2	3.1	2.9	3.2
FRandR	1.4	1.2	0.5	1.0	17.2	0.2	0.2	0.4	0.7	0.2	0.5	0.5	0.3	2.0	2.8	2.1	1.9	2.4	0.8	1.1	2.4	0.1	0.1	0.1

Channels	'TR4-5'	'TR5-6'	'TR6-7'	'TR7-8'	'TR8-9'	'TR9-10'
Ripples	8.9	9.0	1.0	0.4	0.1	0.1
FR	2.6	2.5	2.2	2.4	2.3	2.6
FRandR	0.0	0.0	0.0	0.0	0.0	0.0

Patient 15 ILAE 1

Channels	'TBAL1-2'	'TBAL2-3'	'TBAL3-4'	'TBPL1-2'	'TBPL2-3'	'TBPL3-4'	'TLL1-2'	'TLL2-3'	'TLL3-4'	'TLL4-5'	'TLL5-6'	'TLL9-10'	'TLL10-11'	'TLL11-12'	'TLL12-13'	'TLL13-14'	'TLL17-18'	'TLL18-19'	'TLL19-20'	'TLL20-21'	'TLL21-22'	'TLL25-26'	'TLL26-27'	'TLL27-28'
Ripples	99.2	53.0	22.7	166.8	177.6	75.6	191.8	158.1	123.2	137.3	151.7	66.2	80.8	145.4	115.4	142.4	126.5	197.6	224.8	210.7	206.1	75.5	125.4	130.2
FR	2.6	3.2	7.7	9.7	5.9	2.8	5.9	19.0	19.9	3.7	3.3	3.3	3.5	3.5	3.0	3.0	3.5	3.5	3.3	3.9	2.7	4.1	3.4	5.1
FRandR	0.6	0.3	1.3	1.5	1.5	0.5	2.5	1.5	1.6	0.9	1.3	0.5	0.7	1.1	0.9	1.1	1.1	1.5	1.5	1.4	1.2	0.9	0.8	1.4

Channels	'TLL28-29'	'TLL29-30'	'TLL30-31'	'TLL6-7'	'TLL7-8'	'TLL14-15'	'TLL15-16'	'TLL22-23'	'TLL23-24'	'TLL31-32'
Ripples	153.3	200.9	209.6							
FR	3.5	3.8	3.6							
FRandR	1.2	1.5	1.5							

Patient 16 ILAE 1

Channels	'GL1-2'	'GL2-3'	'GL3-4'	'GL4-5'	'GL5-6'	'GL6-7'	'GL7-8'	'GL9-10'	'GL10-11'	'GL11-12'	'GL12-13'	'GL13-14'	'GL14-15'	'GL15-16'	'GL17-18'	'GL18-19'	'GL19-20'	'GL20-21'	'GL21-22'	'GL22-23'	'GL23-24'	'GL25-26'	'GL26-27'	'GL27-28'
Ripples	26.7	48.1	48.0	43.4	104.5	105.1	129.3	58.4	103.0	96.9	69.0	80.2	88.4	104.8	127.4	77.6	31.7	94.7	108.3	86.7	96.1	80.7	26.0	72.0
FR	2.1	2.7	2.8	2.3	13.4	15.7	8.5	3.4	3.8	4.3	2.6	24.4	26.6	10.8	7.3	3.4	2.3	4.6	9.2	26.0	24.5	4.9	2.8	3.3
FRandR	0.1	0.4	0.5	0.3	5.8	6.8	4.6	0.8	1.3	1.5	0.5	11.5	6.6	3.4	2.5	1.0	0.2	1.3	2.1	4.1	4.0	2.0	0.2	0.9

Channels	'GL28-29'	'GL29-30'	'GL30-31'	'GL31-32'	'TL1-2'	'TL2-3'	'TL3-4'	'TL4-5'	'TL5-6'	'TL6-7'	'TL7-8'	'TL8-9'	'TL9-10'
Ripples	110.0	116.0	85.9	91.7	57.7	49.7	63.7	54.3	48.4	0.3	0.1	0.1	0.1
FR	8.9	10.3	5.0	4.4	64.6	41.4	12.6	5.0	2.6	2.9	2.9	2.6	2.6
FRandR	3.1	4.4	1.9	1.9	35.6	15.3	7.7	2.7	0.7	0.0	0.0	0.0	0.0

Patient 17 ILAE 5

Channels	'FAR1-2'	'FAR2-3'	'FAR3-4'	'FAR4-5'	'FAR5-6'	'FAR6-7'	'FAR7-8'	'FAR9-10'	'FAR10-11'	'FAR11-12'	'FAR12-13'	'FAR13-14'	'FAR14-15'	'FAR15-16'	'FPR1-2'	'FPR2-3'	'FPR3-4'	'FPR4-5'	'FPR5-6'	'FPR6-7'	'FPR7-8'	'FPR9-10'	'FPR10-11'	'FPR11-12'
Ripples	15.8	55.0	94.4	96.6	77.4	96.0	105.0	15.0	33.6	56.2	82.6	120.6	127.6	90.8	111.6	116.4	77.6	45.2	41.2	37.0	57.8	73.4	81.8	66.0
FR	0.0	2.0	6.2	11.0	21.8	25.0	16.0	0.0	2.8	2.4	17.4	16.6	5.0	6.8	5.0	25.8	26.6	9.4	5.0	6.8	3.2	24.2	27.0	36.4
FRandR	0.0	0.6	2.0	0.6	1.0	1.0	0.4	0.0	0.6	0.0	0.8	0.4	0.8	0.6	1.4	1.0	1.2	0.6	0.8	1.4	0.4	2.0	2.8	0.8

Channels	'FPR12-13'	'FPR13-14'	'FPR14-15'	'FPR15-16'	'TR1-2'	'TR2-3'	'TR3-4'	'TR4-5'	'TR5-6'	'TR6-7'	'TR7-8'	'TR8-9'	'TR9-10'
Ripples	67.4	80.8	65.0	81.6	46.6	48.6	38.0	32.6	31.0	34.4	30.2	18.4	3.4
FR	33.8	12.6	19.8	19.8	9.6	9.8	17.4	13.6	3.6	5.4	3.8	2.2	2.4
FRandR	1.0	1.2	1.4	0.6	1.6	1.2	2.0	1.8	0.2	0.8	0.4	0.0	0.0

Patient 18 ILAE 5

Channels	'IHAL1-2'	'IHAL2-3'	'IHAL3-4'	'IHPL1-2'	'IHPL2-3'	'IHPL3-4'	'PLL1-2'	'PLL2-3'	'PLL3-4'	'PLL4-5'	'PLL5-6'	'PML3-4'	'PML4-5'	'PML5-6'	'TL1-2'	'TL2-3'	'TL3-4'	'TL4-5'	'TL5-6'	'TL6-7'	'TL7-8'	'TL8-9'	'TL9-10'	PM1-2	PM2-3
Ripples	31.3	41.4	80.4	162.8	64.2	16.8	139.0	137.3	86.6	65.2	43.0	18.8	34.6	41.1	0.2	1.1	8.3	21.4	19.2	11.5	1.5	3.8	2.1		
FR	2.8	2.4	2.3	7.7	2.5	2.2	20.2	14.1	3.3	4.2	2.6	2.6	2.1	1.8	2.2	4.1	29.4	25.88	4.1	4.1	2.6	2.6	2.8		
FRandR	0.2	0.0	0.6	4.4	0.4	0.1	5.9	7.4	1.3	1.0	0.4	0.1	0.2	0.3	0.0	0.6	1.8	1.4	0.0	0.1	0.0	0.0	0.0		

Patient 19 ILAE 6

Channels	'PL2-3'	'PL3-4'	'PL4-5'	'PL5-6'	'PL6-7'	'PL7-8'	'PL10-11'	'PL11-12'	'PL12-13'	'PL13-14'	'PL14-15'	'PL15-16'	'PL18-19'	'PL19-20'	'PL20-21'	'PL21-22'	'PL22-23'	'PL23-24'	'PL26-27'	'PL27-28'	'PL28-29'	'PL29-30'	'PL30-31'	'PL31-32'
Ripples	34.1	32.8	55.0	55.8	35.5	24.3	38.9	32.2	55.9	71.3	64.3	55.2	44.9	29.5	7.4	16.1	38.8	32.9	45.3	21.6	31.0	32.1	49.8	42.8
FR	1.9	1.8	2.8	3.7	3.5	3.3	2.6	2.7	4.1	22.6	21.5	4.6	2.8	2.3	2.5	3.6	19.4	15.7	2.5	2.5	3.1	7.1	18.8	16.5
FRandR	0.4	0.1	0.8	0.8	0.4	0.4	0.4	0.5	1.4	3.6	3.0	1.4	0.6	0.4	0.0	0.6	11.9	10.2	0.3	0.1	0.8	0.6	4.7	8.2

Channels	'TL1-2'	'TL2-3'	'TL3-4'	'TL4-5'	'TL5-6'	'TL6-7'	'TL7-8'	'TL9-10'	'TL10-11'	'TL11-12'	'TL12-13'	'TL13-14'	'TL14-15'	'TL15-16'
Ripples	46.9	37.6	62.7	109.3	92.8	63.3	42.2	39.2	69.5	60.6	28.0	59.6	66.9	71.3
FR	8.6	9.2	7.6	8.6	6.0	1.2	3.3	10.0	8.4	4.8	3.2	3.6	2.8	2.4
FRandR	1.2	1.2	0.6	0.9	0.9	0.1	0.5	1.3	0.9	0.6	0.7	0.7	0.6	0.5

Patient 20 ILAE 5

Channels	'OTL2-3'	'OTL3-4'	'OTL4-5'	'OTL5-6'	'OTL6-7'	'OTL7-8'	'OTL9-10'	'OTL10-11'	'OTL11-12'	'OTL12-13'	'OTL13-14'	'OTL14-15'	'OTL15-16'	OTL1-2'
Ripples	98.0	105.0	75.8	13.6	56.8	95.4	59.8	56.2	85.9	96.3	65.4	15.8	57.4	
FR	3.7	3.5	3.2	2.9	4.4	5.7	6.3	8.6	7.2	3.5	2.7	3.0	2.8	
FRandR	1.2	1.0	0.6	0.4	0.9	1.5	1.3	1.6	1.2	0.9	0.5	0.4	0.8	
